# Exacerbation of hepatic injury during rodent malaria by myeloid-related protein 14

**DOI:** 10.1371/journal.pone.0199111

**Published:** 2018-06-14

**Authors:** Haruka Mizobuchi, Wataru Fujii, Shoko Isokawa, Kanna Ishizuka, Yihan Wang, Sayoko Watanabe, Chizu Sanjoba, Yoshitsugu Matsumoto, Yasuyuki Goto

**Affiliations:** 1 Laboratory of Molecular Immunology, Graduate School of Agricultural and Life Sciences, The University of Tokyo, Bunkyo-ku, Tokyo, Japan; 2 Laboratory of Applied Genetics, Graduate School of Agricultural and Life Sciences, The University of Tokyo, Bunkyo-ku, Tokyo, Japan; National Institutes of Health, UNITED STATES

## Abstract

Hepatic dysfunction is one of the clinical features in severe malaria. However, the mechanism of hepatic injury during malaria is still unknown. Myeloid-related protein (MRP) 14 is abundantly expressed by myeloid cells and involved in various inflammatory diseases. We previously reported that serum MRP14 is elevated in mice infected with *Plasmodium berghei* ANKA. In order to verify whether extracellular MRP14 is involved in the pathology of hepatic injury during rodent malaria, we intravenously administrated recombinant MRP14 (rMRP14) to mice infected with *P*. *berghei* ANKA. The administration of rMRP14 did not affect parasite number or hematocrit. On the other hand, the hepatic injury was exacerbated in rMRP14-treated mice, and their serum concentration of hepatic enzymes increased significantly more than PBS-treated controls. Immunohistochemical analysis of the liver showed that more MRP14^+^ macrophages accumulated in rMRP14-treated mice than PBS-treated controls after infection. The administration of rMRP14 also promotes the up-regulation of pro-inflammatory molecules in the liver, such as iNOS, IL-1β, IL-12, and TNF-α. Even in the absence of Plasmodium infection, administration of rMRP14 could induce the accumulation of MRP14^+^ macrophages and up-regulation of the pro-inflammatory molecules in the liver of naïve mice. The results indicate that MRP14 promotes the accumulation of MRP14^+^ cells and the up-regulation of pro-inflammatory molecules and NO, which amplify inflammatory cascade leading to hepatic injury. In conclusion, MRP14 is a one of key molecules for liver inflammation during rodent malaria.

## Introduction

Hepatic dysfunction is one of the common clinical features in severe malaria patients. Severe liver dysfunction occurs occasionally in severe malaria in association with multi-organ failure and poor prognosis [1–3]. In adult non-immune patients in South-East Asia and India, jaundice and liver dysfunction occur in up to 50% of cases in severe malaria, almost always as part of multi-organ disease [4]. Elevations of liver cytoplasmic enzymes are common, including raised aspartate aminotransferase (AST), alanine aminotransferase (ALT) and alkaline phosphatase [2,3]. Histopathological examination of liver biopsies of severe malaria patients showed that dilated sinusoids, parasitized red blood cell (pRBC) sequestration within hepatic sinusoids and adhesion of pRBCs to sinusoidal endothelial cells, and the retention of malaria pigment, accompanied with hepatocyte swelling and necrosis, host macrophage infiltration and focal centrilobular hepatic necrosis [1,4–6]. It is reported that the degree of jaundice, hepatomegaly and liver enzyme abnormalities correlates with the overall parasite load in the body, and the sequestration of pRBCs in the liver was quantitatively associated with liver weight, serum bilirubin and AST levels [7].

Rodent malaria model using BALB/c mice and lethal *Plasmodium berghei* (Pb) ANKA strain shows clinical manifestations as parasitemia, anemia, splenomegaly and hepatic injury, which are also observed in human severe malaria patients as described above. Also, in histopathological analysis of the liver of the mouse model, vasodilatation, remarkable macrophage infiltration, and necrosis of hepatocytes are observed [8–11]. Previous murine studies have shown that IL-12 and IFN-γ have a pivotal role in liver injury caused by *P*. *berghei*. Yoshimoto *et al*. revealed that the lethal *P*. *berghei* NK65 infection induces IL-12 production and the cytokine is involved in the pathogenesis of liver injury via IFN-γ production rather than the protection [9]. Also, in the study by Adachi *et al*., it is demonstrated that the liver injury induced by *P*. *berghei* NK65 infection of mice induces activation of the toll-like receptor (TLR)-MyD88 signaling pathway which results in IL-12 production and activation of the perforin-dependent cytotoxic activities of MHC-unrestricted hepatic lymphocytes [10]. These studies suggest that the hepatic injury induced by *P*. *berghei* is caused by the local production of cytokines that activates inflammatory cells in the liver. However, the mechanism of hepatic injury during *Plasmodium* species infection is not well elucidated.

Myeloid-related protein (MRP) 14 has been characterized as an inflammation-related protein [12–14]. MRP14, which is also known as S100A9, belongs to the S100 calcium-binding protein family and can form the heterodimer with MRP8, which is also known as S100A8 [15–17]. These proteins are expressed by neutrophils and monocytes [15] and are also known as markers of inflammatory macrophages. The previous studies have revealed that MRP14 play a pivotal role in the pathogenesis of various inflammatory disorders. In inflammatory diseases such as rheumatoid arthritis, psoriatic arthritis, and coronary syndromes, the accumulation of cells expressing MRP14 is observed at inflammatory sites [18–20]. In addition, the protein is secreted by the inflammatory cells when activated [21]. Actually, MRP14 in serum is elevated in various diseases including rheumatoid arthritis [19], coronary syndromes[22], and psoriatic arthritis [23]. Moreover, some studies suggest the function of MRP14, not only as biomarkers but also inflammation mediators [12,24]. Those results suggest that extracellular MRP14 is involved in the inflammation accompanied with the accumulation of MRP14^+^ macrophages. For malaria, there is a report that elevated serum MRP14 levels in falciparum malaria patients correlated with an elevated parasite load [25]. We previously reported the accumulation of macrophages expressing MRP14 in the spleen of BALB/c mice infected with *P*. *berghei* ANKA [26]. Immunohistochemical analysis showed that the locations of MRP14^+^ macrophages were similar to those of MRP8^+^ macrophages, which indicates that MRP14^+^ macrophages also express MRP8. In addition, we also revealed the MRP14 level in the plasma was also elevated during Pb-infection compared with uninfected controls. However, the role of MRP14 in hepatic injury during rodent malaria has been unclear.

Taken together, it has been hypothesized that the accumulation of MRP14^+^ cells in the tissue is associated with an increase in MRP14 in serum during malaria and that the extracellular MRP14 is involved in hepatic injury. In the present study, in order to verify whether extracellular MRP14 is involved in the pathology of hepatic injury during malaria, we intravenously administrated MRP14 to mice infected with *P*. *berghei* ANKA.

## Results

### Enhanced serum MRP14 level dependent on hepatic injury during rodent malaria

**Fig 1:**
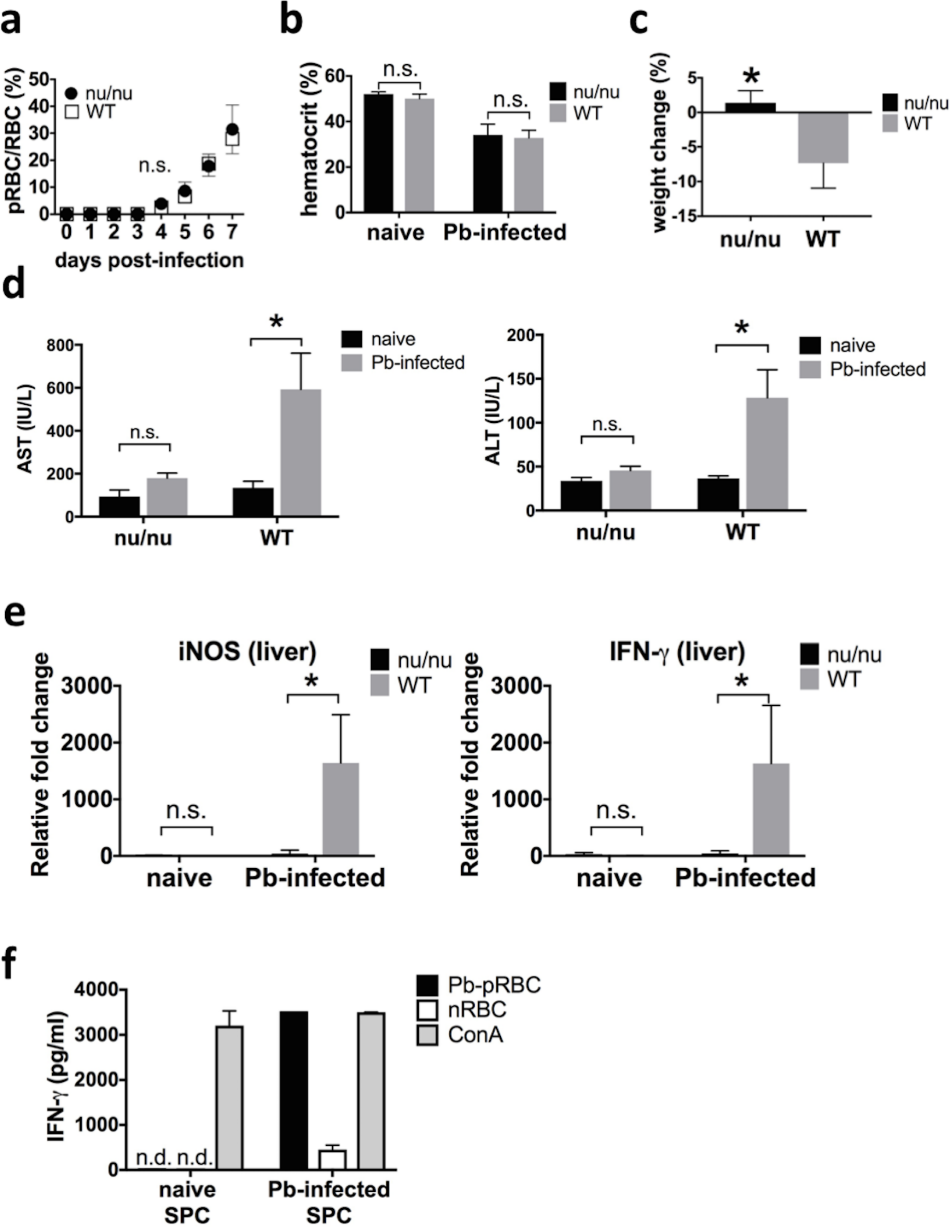
Hepatic injury regulated by T cells during rodent malaria. (a) Kinetics of pRBC rate in peripheral blood after infection of 10^6^ pRBCs in *nu/nu* mice and WT mice (n = 5). (b) Hematocrit in *nu/nu* mice and WT mice after Pb-infection (n = 5). (c) The body weight change of *nu/nu* mice and WT mice after Pb-infection (n = 5). (d) Serum concentration of AST (left) and ALT (right) of *nu/nu* mice and WT mice after Pb-infection (n = 5). (e) mRNA expression of iNOS (left) and IFN-γ (right) in the liver of *nu/nu* mice and WT mice treated with LPS (n = 5). (f) IFN-γ produced by Pb-pRBC antigen-specific T cells. Splenocytes (5 x 10^6^ cells/ml) of Pb-infected mice or naïve mice were stimulated by Pb-pRBC (1 x 10^8^ cells/ml), nRBC (1 x 10^8^ cells/ml) or conA (3 µg/ml) and the concentration of IFN-γ secreted in supernatant was measured by ELISA. Graphs show mean and SD of each group. Data are representative of two independent experiments. ^*^P < 0.05; n.s., not significant.

*P*. *berghei* ANKA causes fatal infection in BALB/cA mice; infection with 10^6^ pRBCs kills mice in 8 days with an associated 60–80% parasitemia level (data not shown). In this study, therefore, pRBC rate was monitored every day, and mice were sacrificed at day 7 post-infection to collect blood and tissues. In order to elucidate whether T cells are involved in hepatic injury during malaria, T cell-deficient *nu/nu* mice were infected with Pb-pRBCs. There was no significant difference in pRBC rate and hematocrit between *nu/nu* mice and WT mice. On day 7 post-infection, the mean ± SD of pRBC rate was 31.4 ± 8.1% in *nu/nu* mice and 27.8 ± 4.9% in WT mice (Fig 1A), and the mean ± SD of hematocrit was 34.0 ± 4.3% in *nu/nu* mice and 32.8 ± 3.0% in WT mice (Fig 1B). On the other hand, WT mice lost significantly more body weight than *nu/nu* mice during infection. On day 7 post-infection, the mean ± SD of body weight change was 1.4 ± 1.6% in *nu/nu* mice and -7.3 ± 3.2% in WT mice (Fig 1C). The concentrations of serum AST and ALT were not increased in *nu/nu* mice during Pb-infection. (Fig 1D). In *nu/nu* mice, there was no difference in serum concentration of AST (naïve: 93.8 ± 27.1 IU/L, Pb-infected: 179.4 ± 21.2 IU/L) and ALT (naïve: 33.8 ± 3.3 IU/L, Pb-infected: 45.6 ± 04.3 IU/L) between naïve and Pb-infected mice, whereas the serum concentrations of AST and ALT were significantly higher after Pb-infection in WT mice (naïve: 134.4± 27.7IU/L, Pb-infected: 593.2 ± 150.3 IU/L for AST; naïve: 36.6 ± 2.6 IU/L, Pb-infected: 128.4 ± 28.4 IU/L for ALT). The mRNA expression of iNOS and IFN-γ in the liver was significantly higher in WT mice than in *nu/nu* mice after Pb-injection (Fig 1E). In *nu/nu* mice, the expression of iNOS and IFN-γ in the liver was low and not different between naïve mice and Pb-infected mice. Splenocyte assay showed that antigen recall with Pb-pRBCs induced IFN-γ production by splenocytes (Fig 1F). While IFN-γ produced by naïve splenocytes stimulated with Pb-pRBCs or nRBCs was below the detection limit, splenocytes from Pb-infected mice produced higher IFN-γ when stimulated with Pb-pRBCs than when stimulated with nRBCs.

**Fig 2:**
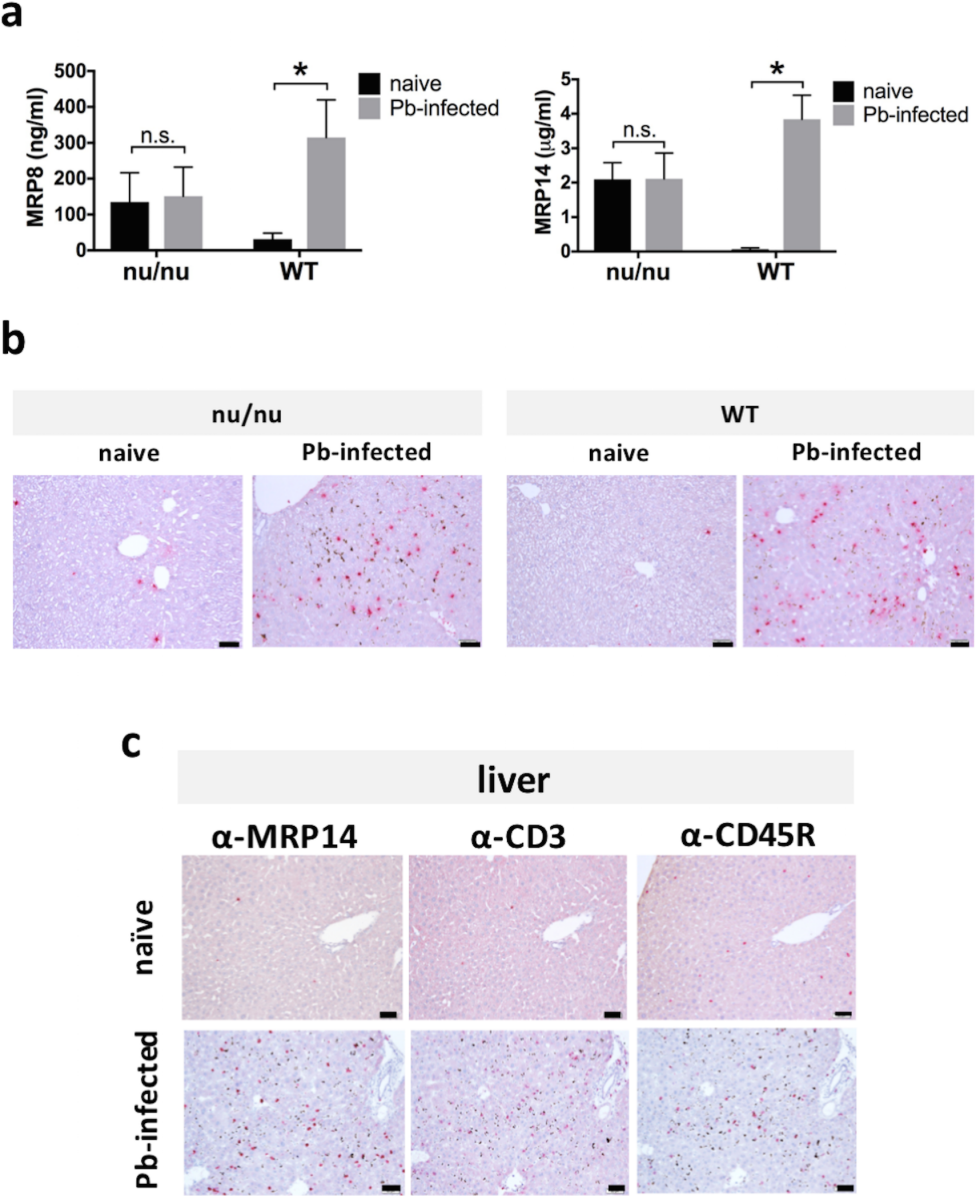
Increase of extracellular MRP14 depend on T cells during rodent malaria. (a) Serum concentration of MRP8 (left) and MRP14 (right) of *nu/nu* mice and WT mice after Pb-infection (n = 5). (b) The accumulation of MRP14^+^ cells in the liver of *nu/nu* mice and WT mice after Pb-infection. RP, red pulp; WP, white pulp. Bars; 50 µm. (c) The accumulation of MRP14^+^ cells, CD3^+^ cells and CD45^+^ cells in the liver of Pb-infected mice. RP, red pulp; WP, white pulp. Bars; 50 µm. Graphs show mean and SD of each group. Data are representative of two independent experiments. ^*^P < 0.05; n.s., not significant.

The concentrations of serum MRP8 and MRP14 showed similar tendency to that of serum AST and ALT. The increase levels of serum MRP8 and MRP14 were significantly lower in *nu/nu* mice than WT mice (Fig 2A). In *nu/nu* mice, there was no difference in serum concentration of MRP8 (naïve: 135.4 ± 72.5 ng/ml, Pb-infected: 151.3 ± 72.3 ng/ml) and MRP14 (naïve: 2.1 ± 0.4 µg/ml, Pb-infected: 2.1 ± 0.7 µg/ml) between naïve mice and Pb-infected mice (Fig 2A), while serum concentration of MRP8 and MRP14 was significantly higher after Pb-infection in WT mice (naïve: 30.9 ± 15.4 ng/ml, Pb-infected: 315.2 ± 93.5 ng/ml for MRP8; naïve: 0.06 ± 0.04 µg/ml, Pb-infected: 3.8 ± 0.6 µg/ml for MRP14). In immunohistochemical analysis, the accumulation of MRP14^+^ cells in the liver was observed in both of *nu/nu* mice and WT mice after infection (Fig 2B). The accumulation level and staining pattern of MRP8^+^ cells and MRP14^+^ cells were similar (data not shown). It was shown that not only MRP14^+^ and MRP8^+^ cells but also CD3^+^ cells and CD45R^+^ cells were accumulated in the liver after Pb-infection (Fig 2C).

### Macrophage activation induced by recombinant MRP14

**Fig 3:**
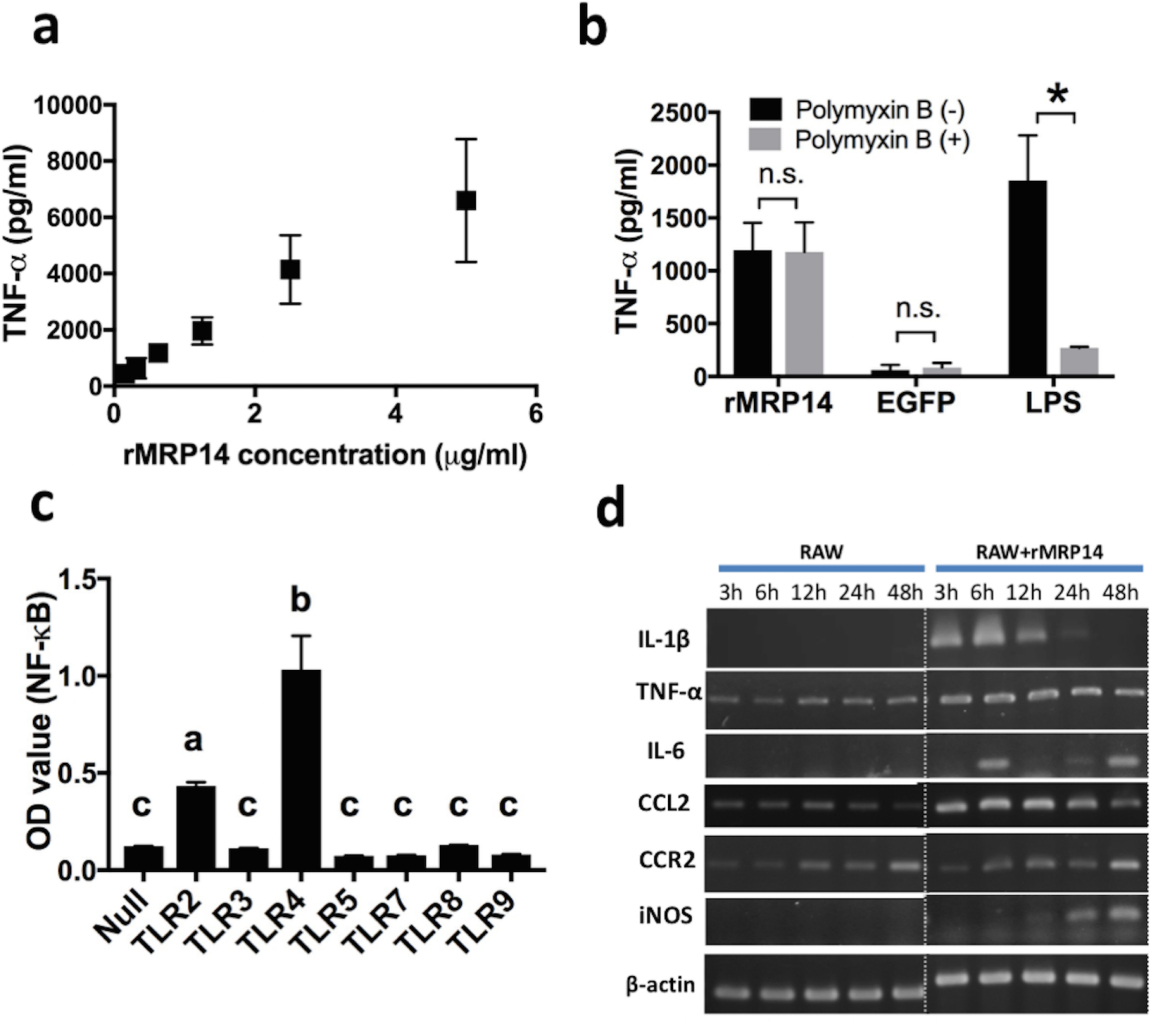
Macrophage activation induced by rMRP14. (a) Increase of TNF-α secretion dependent on rMRP14 concentration in RAW264.7 cells. RAW264.7 cells (2 x 10^5^ cells/ml) were stimulated by rMRP14 (0.15–5 µg/ml) and the concentration of secreted TNF-α in supernatant was measured by ELISA. (b) The increase of TNF-α by rMRP14 could not be blocked by addition of polymyxin B. RAW264.7 cells (2 x 10^5^ cells/ml) were stimulated by rMRP14 (100 ng/ml), EGFP (100 ng/ml) or LPS (2.5 ng/ml) incubated with or without polymyxin B (50 μg/ml), which is a LPS inhibitor. EGFP is recombinant proteins with polyhistidine-tag, which was used as a negative control. (c) MRP14 as an agonist of TLR2 and TLR4. HEK293 cells transfected with murine TLR gene were stimulated by rMRP14 (5 µg/ml) and SEAP induced by NF-*κ*B activation through TLR was measured. Different letters on the bars represent statistical difference between groups. (d) mRNA expression of IL-1β, TNF-α, IL-6, CCL2, CCR2, iNOS and β-actin in RAW264.7 cells (2 x 10^5^ cells/ml) stimulated by rMRP14 (5 µg/ml). Graphs show mean and SD of triplicates. Data are representative of three independent experiments. ^*^P < 0.05; n.s., not significant.

RAW264.7 cells were stimulated by recombinant MRP14 (rMRP14) and the concentration of secreted TNF-α in the supernatant was measured by ELISA. TNF-α was induced after stimulation with rMRP14. Moreover, the increase of TNF-α concentration was proportional to the increase of MRP14 concentration (Fig 3A). The increase of TNF-α induced by rMRP14 was not blocked by addition of polymyxin B (Fig 3B). In contrast, polymyxin B efficiently blocked TNF-α induction by LPS. The concentration of TNF-α remained low after stimulation with recombinant EGFP, which was used as an irrelevant 6xHis-tagged recombinant protein. In order to reveal activation of TLR signal pathway by rMRP14, HEK293 cells transfected with a murine TLR gene were stimulated by rMRP14 and secreted embryonic alkaline phosphatase (SEAP) induced by NF-*κ*B activation through TLR was measured (Fig 3C). HEK293 cells expressing TLR4 showed significantly strong induction of SEAP after stimulation with MRP14. In addition, HEK293 cells expressing TLR2 also showed a strong induction of SEAP after stimulation with rMRP14. In contrast, HEK 293 cells expressing TLR3, 5, 7, 8 or 9 showed low level of SEAP as HEK293/Null cells. RT-PCR analysis showed that rMRP14 induced the mRNA expression of IL-1β, TNF-α, IL-6, CCL2 and iNOS in RAW264.7 cells (Fig 3D). The expression of IL-1β, TNF-α, IL-6 and CCL2 was promoted at 3–6 hr after stimulation, and the expression of iNOS was promoted at 24 hr after stimulation.

### Exacerbation of hepatic injury by rMRP14 during rodent malaria

**Fig 4:**
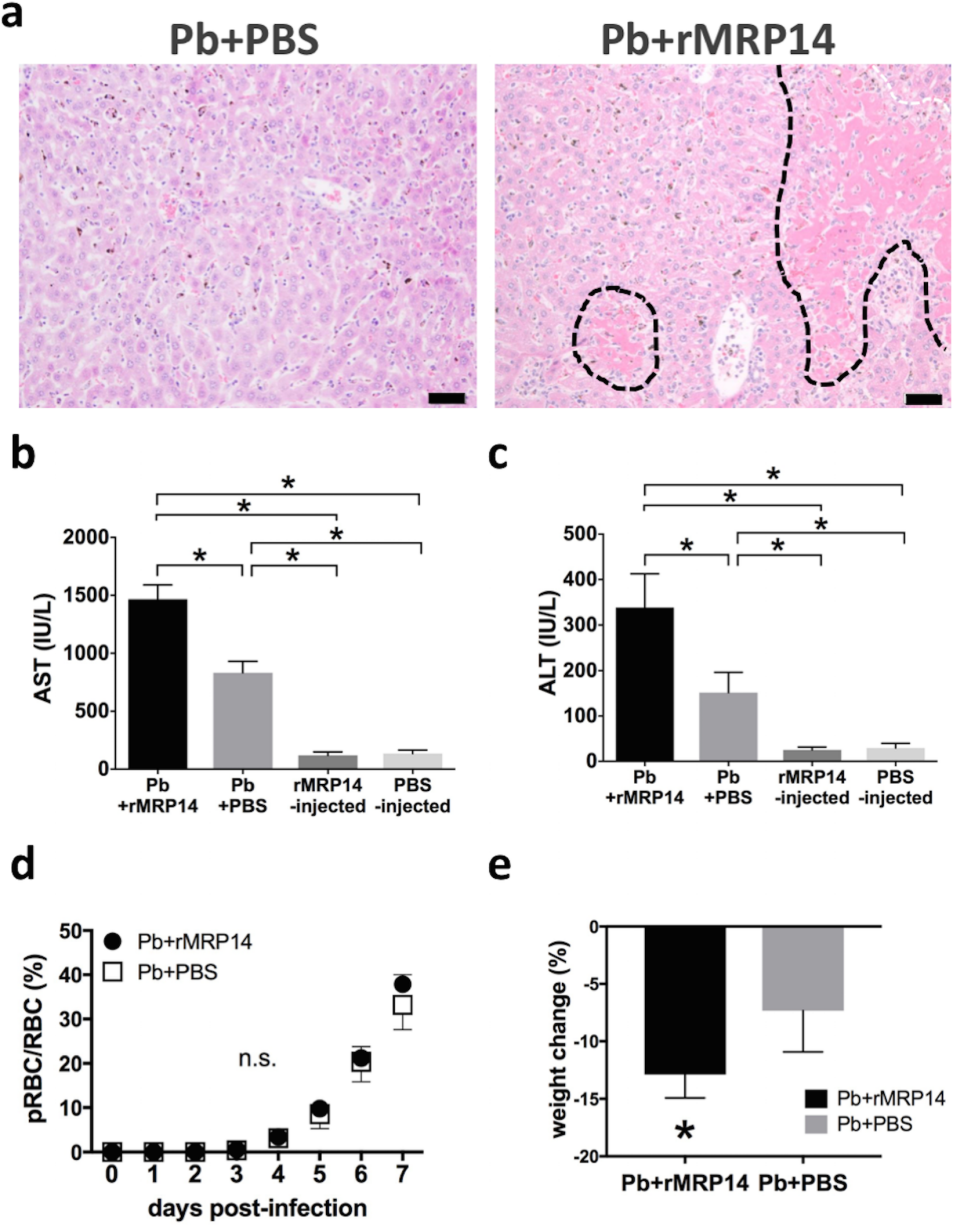
Exacerbation of hepatic injury by rMRP14 during rodent malaria. (a) Histopathology of the liver of mice injected with rMRP14 or PBS after Pb-infection analyzed by HE staining. The area surrounded by dotted line shows focal necrosis. Bar; 50 µm. (b)(c) Serum concentration of AST (b) and ALT (c) of mice injected with rMRP14 or PBS after Pb-infection (n = 5). (d) No effect of rMRP14 on parasite proliferation. Kinetics of pRBC rate in peripheral blood after infection of 10^6^ pRBCs in rMRP14-injected mice and PBS-injected controls (n = 5). (e) The body weight change of mice injected with rMRP14 or PBS after Pb-infection (n = 5). Graphs show mean and SD of each group. Data are representative of two independent experiments. ^*^P < 0.05; n.s., not significant.

Histopathological analysis of the liver showed that hepatic injury was exacerbated by rMRP14 during Pb-infection. In the liver of both rMRP14-injected and PBS-injected mice, vasodilatation, remarkable cellular infiltration, and degeneration of hepatocytes were observed after Pb-infection. In the liver of rMRP14-injected mice, a lot of focal necrosis areas were observed, whereas few focal necrosis areas were observed in PBS-injected mice after Pb-infection (Fig 4A). Serum concentration of AST and ALT increased significantly higher in rMRP14-injected mice than in PBS-injected controls at day 7 after Pb-injection (Fig 4B and 4C). On day 7 post-infection, the concentration of serum AST was 1,467 ± 110 IU/L in MRP14-injected mice and 833 ± 89 IU/L in PBS-injected mice, and the concentration of serum ALT was 338 ± 66 IU/L in rMRP14-injected mice and 151 ± 40 IU/L in PBS-injected mice. In the absence of Pb-infection, no significant difference was observed in serum AST (MRP14-injected, 119 ± 27 IU/L; PBS-injected, 134 ± 28 IU/L) and ALT (rMRP14-injected, 25 ± 6 IU/L; PBS-injected, 29 ± 10 IU/L) between the rMRP14-injected mice and PBS-injected mice. On the other hand, there was no significant difference in pRBC rate and hematocrit between the rMRP14-injected mice and PBS-injected mice. On day 7 post-infection, the mean ± SD of pRBC rate was 37.8 ± 2.0% in rMRP14-injected mice and 33.2 ± 5.0% in PBS-injected mice (Fig 4D). rMRP14-injected mice lost significantly more body weight than PBS-injected mice during Pb-infection. On day 7 post-infection, the mean ± SD of body weight change was -12.9 ± 1.8% in rMRP14-injected mice and -7.3 ± 3.2% in PBS-injected mice (Fig 4E).

### The accumulation of MRP8^+^ and MRP14^+^ cells promoted by rMRP14 during rodent malaria

**Fig 5:**
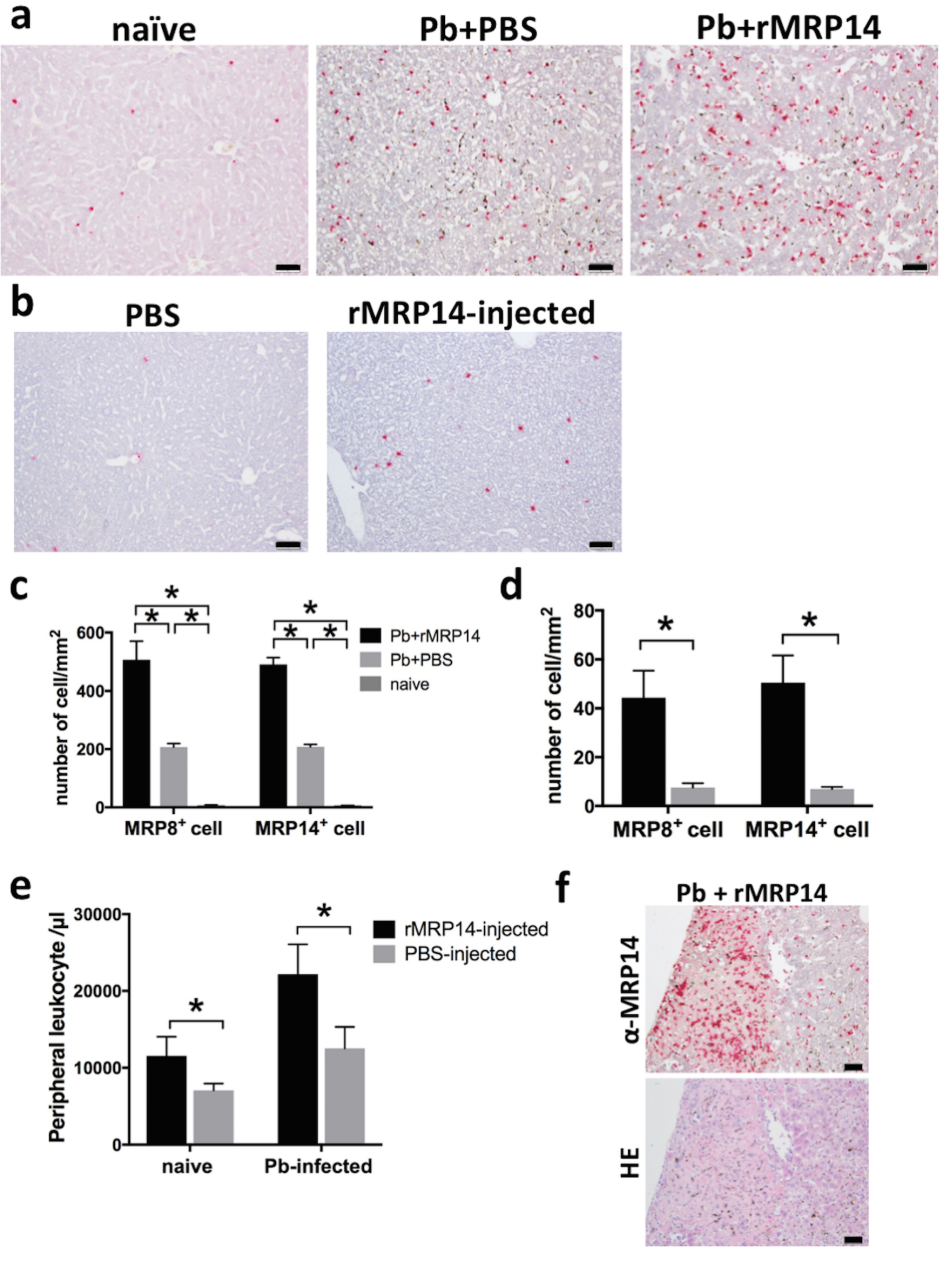
The accumulation of MRP14^+^ and MRP8^+^ cells promoted by rMRP14 in the liver during rodent malaria. (a) The accumulation of MRP14^+^ cells in the liver of mice injected with rMRP14 or PBS after Pb-infection. Bars; 50 µm. (b)The accumulation of MRP14^+^ cells in the liver of rMRP14-injected naïve mice and PBS-injected controls. Bars; 50 µm. (c) The number of accumulated MRP8^+^ and MRP14^+^ cells in the liver of mice injected with rMRP14 or PBS after Pb-infection (n = 5). (d) The number of accumulated MRP8^+^ and MRP14^+^ cells in the liver of naïve mice injected with rMRP14 or PBS (n = 5). (e) The number of peripheral blood leukocytes of mice injected with rMRP14 or PBS after Pb-infection (n = 5). (f) The accumulation of MRP14^+^ cells in focal necrosis area of the liver in mice injected with rMRP14 or PBS after Pb-infection. Bars; 50 µm. Graphs show mean and SD of each group. Data are representative of two independent experiments. ^*^P < 0.05; n.s., not significant.

Immunohistochemical analysis showed that MRP8^+^ and MRP14^+^ cells were accumulated in the liver at day 7 after Pb-infection, and the accumulation of MRP8^+^ and MRP14^+^ cells was amplified by rMRP14-injection (Fig 5A). The quantitative analysis demonstrated that the number of the MRP14^+^ and MRP8^+^ cells in the liver was significantly higher in rMRP14-injected mice (MRP8^+^ cells: 507 ± 57 cells/mm^2^, MRP14^+^ cells: 491 ± 21 cells/mm^2^) than PBS-injected control mice (MRP8^+^ cells: 207 ± 11 cells/mm^2^, MRP14^+^ cells: 208 ± 7.1 cells/mm^2^) after Pb-infection (Fig 5C). Even in the absence of Pb-infection, the accumulation of MRP14^+^ and MRP8^+^ cells was induced by rMRP14 itself (Fig 5B). The quantitative analysis demonstrated that the number of the MRP8^+^ and MRP14^+^ cells in the liver was significantly higher in rMRP14-injected mice (MRP8^+^ cells: 44 ± 10 cells/mm^2^, MRP14^+^ cells: 51 ± 10 cells/mm^2^) than PBS-injected control mice (MRP8^+^ cells: 7.6 ± 1.5 cells/mm^2^, MRP14^+^ cells: 7.0 ± 0.7 cells/mm^2^) (Fig 5D). The number of peripheral leukocytes was also significantly higher in rMRP14-injected naïve mice (11,560 ± 2,241 cells/µl) than in PBS-injected naïve mice (7,080 ± 803 cells/µl) (Fig 5E). Though the peripheral leukocytes increased after infection in both mouse groups, the number of peripheral leukocytes was significantly higher in rMRP14-injected mice (22,180 ± 3,468 cells/µl) than in PBS-injected mice (12,540 ± 2,484 cells/µl) on day 7 post-infection (Fig 5E). In the liver of rMRP14-injected mice, it was observed that the more MRP14^+^ and MRP8^+^ cells were accumulated in focal necrosis areas than in other areas (Fig 5F).

### The expression of pro-inflammatory molecules promoted by rMRP14 in the liver during rodent malaria

**Fig 6:**
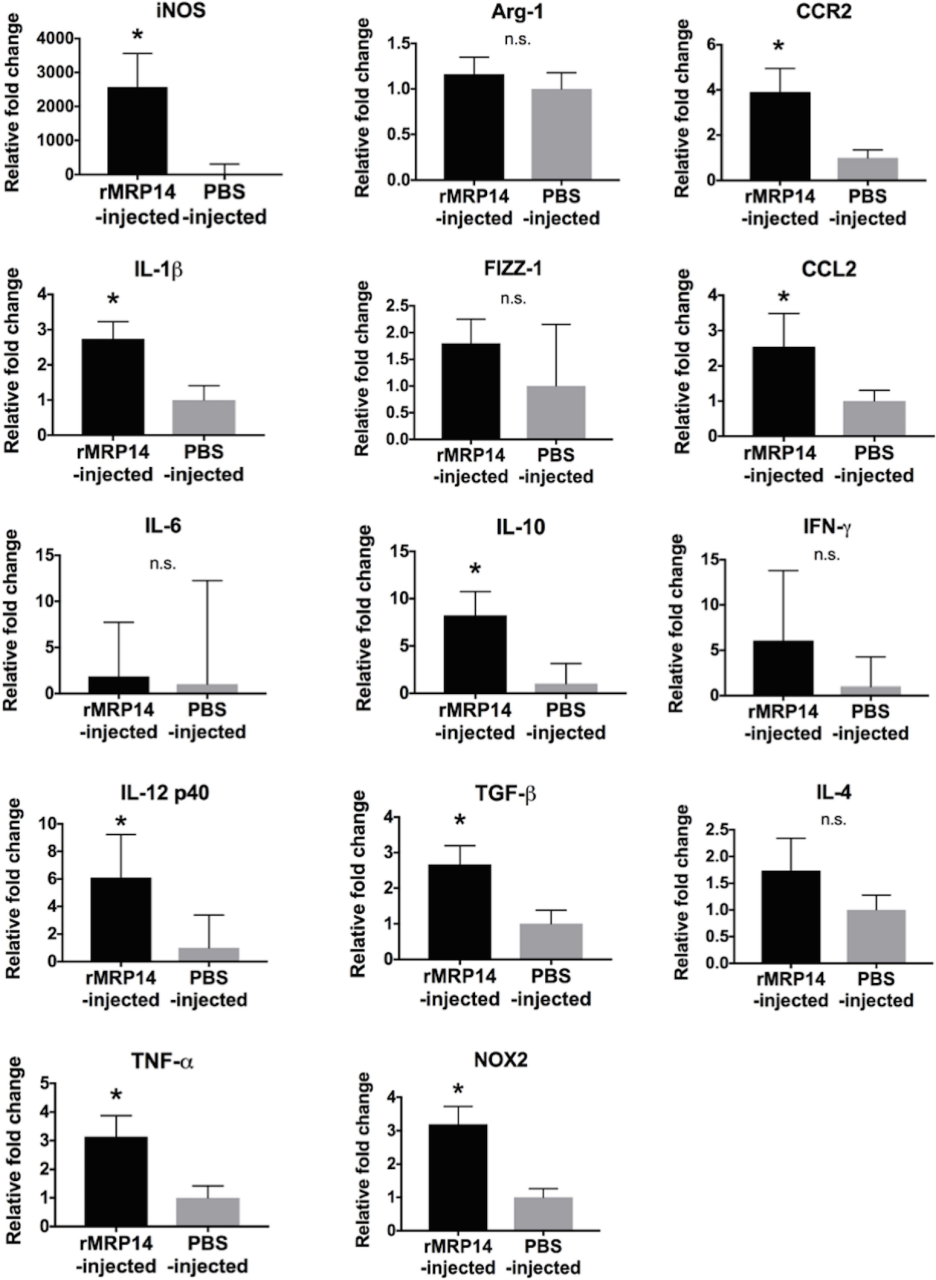
The expression of pro-inflammatory molecules promoted by rMRP14 injection. mRNA expression of iNOS, IL-1β, IL-6, IL-12 p40, TNF-α, Arg-1, FIZZ-1, IL-10, TGF-β, CCR2, CCL2, IFN-γ and IL-4 in the liver of mice injected with rMRP14 or PBS (n = 5). Graphs show mean and SD of each group. Data are representative of two independent experiments. ^*^P < 0.05; n.s., not significant.

**Fig 7:**
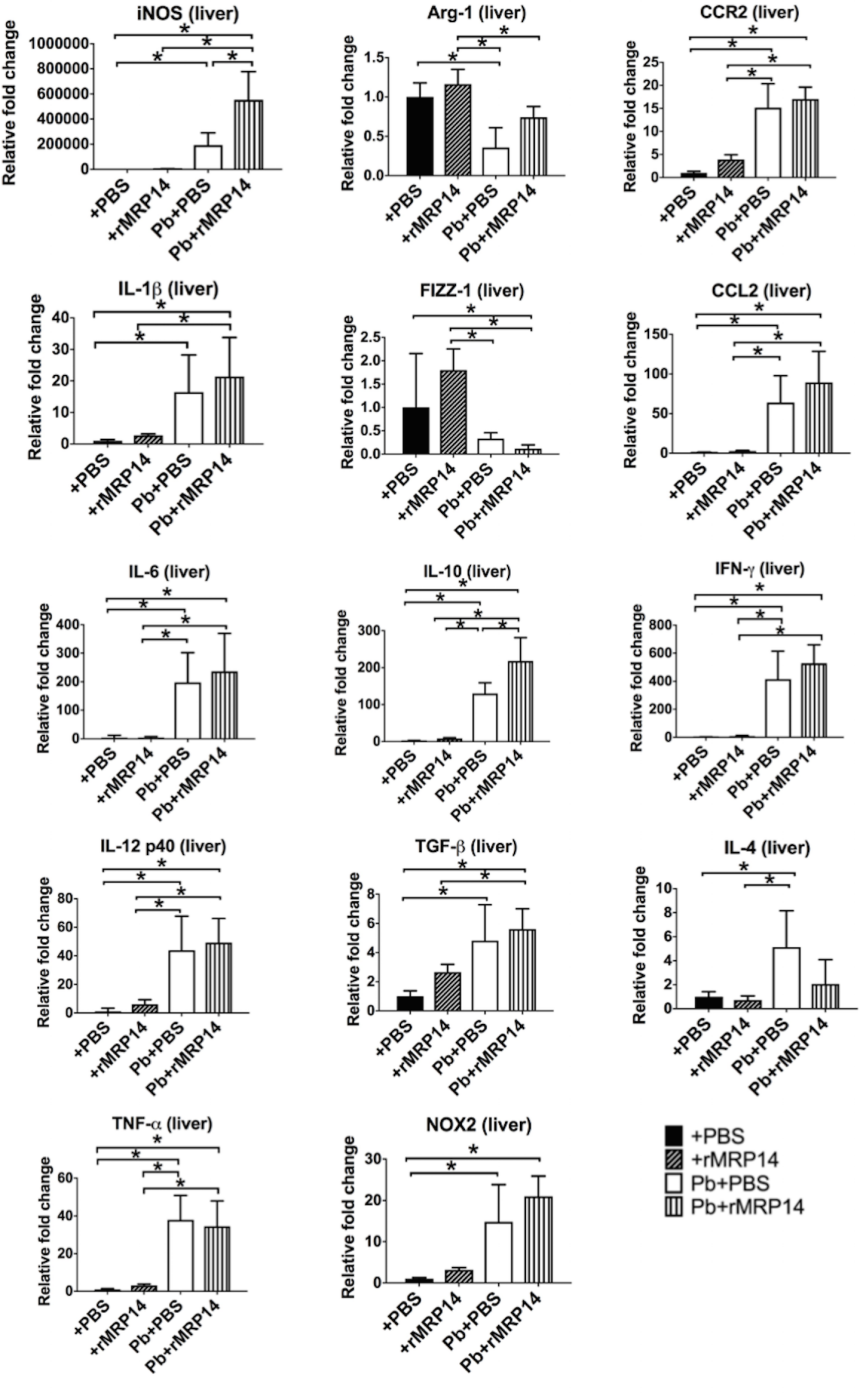
The expression of iNOS and pro-inflammatory molecules in the liver promoted by rMRP14 during rodent malaria. mRNA expression of iNOS, IL-1β, IL-6, IL-12 p40, TNF-α, Arg-1, FIZZ-1, IL-10, TGF-β, NOX2, CCR2, CCL2, IFN-γ and IL-4 in the liver of mice injected with rMRP14 or PBS after Pb-infection (n = 5). Graphs show mean and SD of each group. Data are representative of two independent experiments. ^*^P < 0.05; n.s., not significant.

Immunomodulatory effects of rMRP14 in vivo were examined in the presence/absence of Pb-infection by looking at mRNA levels of pro-inflammatory molecules. Quantitative RT-PCR analysis showed that the expression of pro-inflammatory molecules in the liver was promoted by rMRP14 in the absence of Pb-infection (Fig 6). The expression of iNOS, IL-1β, IL-12 p40, TNF-α, IL-10, TGF-β, NOX2, CCR2 and CCL2 was significantly up-regulated in the liver by MRP14. On day 7 of Pb-infection, the expression of iNOS, IL-1β, IL-6, IL-12 p40, TNF-α, IL-10, TGF-β, NOX2, CCR2, CCL2, IFN-γ and IL-4 was significantly up-regulated, and the expression of Arg-1 and FIZZ-1 was down-regulated in the liver of the infected mice compared with naïve mice (Fig 7). In the presence of Pb-infection, only the expression of iNOS was higher in rMRP14-injected mice than PBS-injected mice.

### Hepatic injury independent of MRP14 during rodent malaria

**Fig 8:**
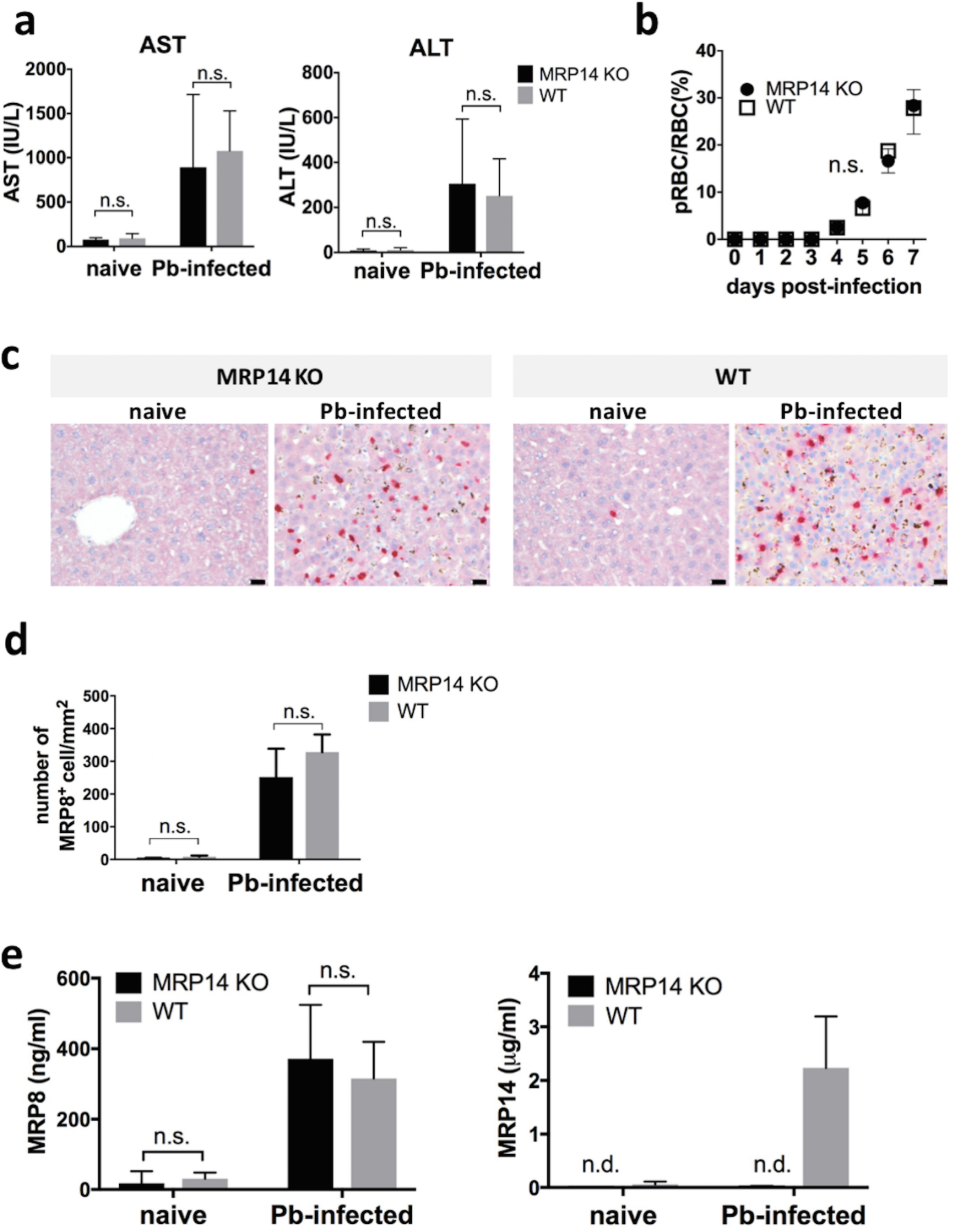
The accumulation of MRP8^+^ cells independent of MRP14 in the liver during rodent malaria. (a) Serum concentration of AST (left) and ALT (right) of MRP14-KO mice and WT mice after Pb-infection (n = 5). (b) No effect of MRP14 deficiency on parasite proliferation. Kinetics of pRBC rate in peripheral blood after infection of 10^6^ pRBCs in MRP14-KO mice and WT mice (n = 5). (c) The accumulation of MRP8^+^ cells in the liver of MRP14-KO mice and WT mice after Pb-infection. Bars; 50 µm. (d) The number of accumulated MRP8^+^ cells in the liver of MRP14-KO mice and WT mice after Pb-infection (n = 5). (e) Serum concentration of MRP8 (left) and MRP14 (right) of MRP14-KO mice and WT mice after Pb-infection (n = 5). Graphs show mean and SD of each group. Data are representative of two independent experiments. ^*^P < 0.05; n.s., not significant.

In order to verify whether MRP14 deficiency affects the pathology during rodent malaria, MRP14-KO mice were infected with Pb-pRBCs. In histopathological analysis of the liver, no pathological difference was observed between MRP14-KO mice and WT mice after Pb-infection. In the liver of Pb-infected mice, vasodilatation and remarkable cellular infiltration were observed in both MRP14-KO and WT mice. Also, serum concentration of AST and ALT increased significantly at day 7 after Pb-infection in both of MRP14-KO mice and WT mice (Fig 8A). No significant difference was observed in serum AST (MRP14-KO, 894 ± 734 IU/L; WT, 1,077 ± 405 IU/L) and ALT (MRP14-KO, 306 ± 257 IU/L; WT, 252 ± 147 IU/L) between MRP14-KO mice and WT mice after Pb-injection. There was no significant difference in pRBC rate between MRP14-KO mice and WT mice. On day 7 post-infection, the mean ± SD of pRBC rate was 28.4 ± 3.0% in MRP14-KO mice and 27.8 ± 5.0% in WT mice (Fig 8B).

Immunohistochemical analysis showed that MRP8^+^ cells were similarly accumulated in the liver at day 7 after Pb-infection in both of MRP14-KO mice and WT mice (Fig 8C). The quantitative analysis demonstrated that the number of the MRP8^+^ cells in the liver was comparable between MRP14-KO mice (252 ± 78 cells/mm^2^) and WT mice (328 ± 48 cells/mm^2^) (Fig 8D). Serum concentration of MRP8 increased significantly at day 7 after Pb-injection in MRP14-KO mice (naïve: 17.5 ± 3.1 ng/ml, Pb-infected: 371.1 ± 137 ng/ml), and the concentration of serum MRP8 was not different between MRP14-KO mice (371.1 ± 137 µg/ml) and WT mice (315.2 ± 93.5 µg/ml) after Pb-infection (Fig 8E). Serum MRP14 was not detected in MRP14-KO mice.

**Fig 9:**
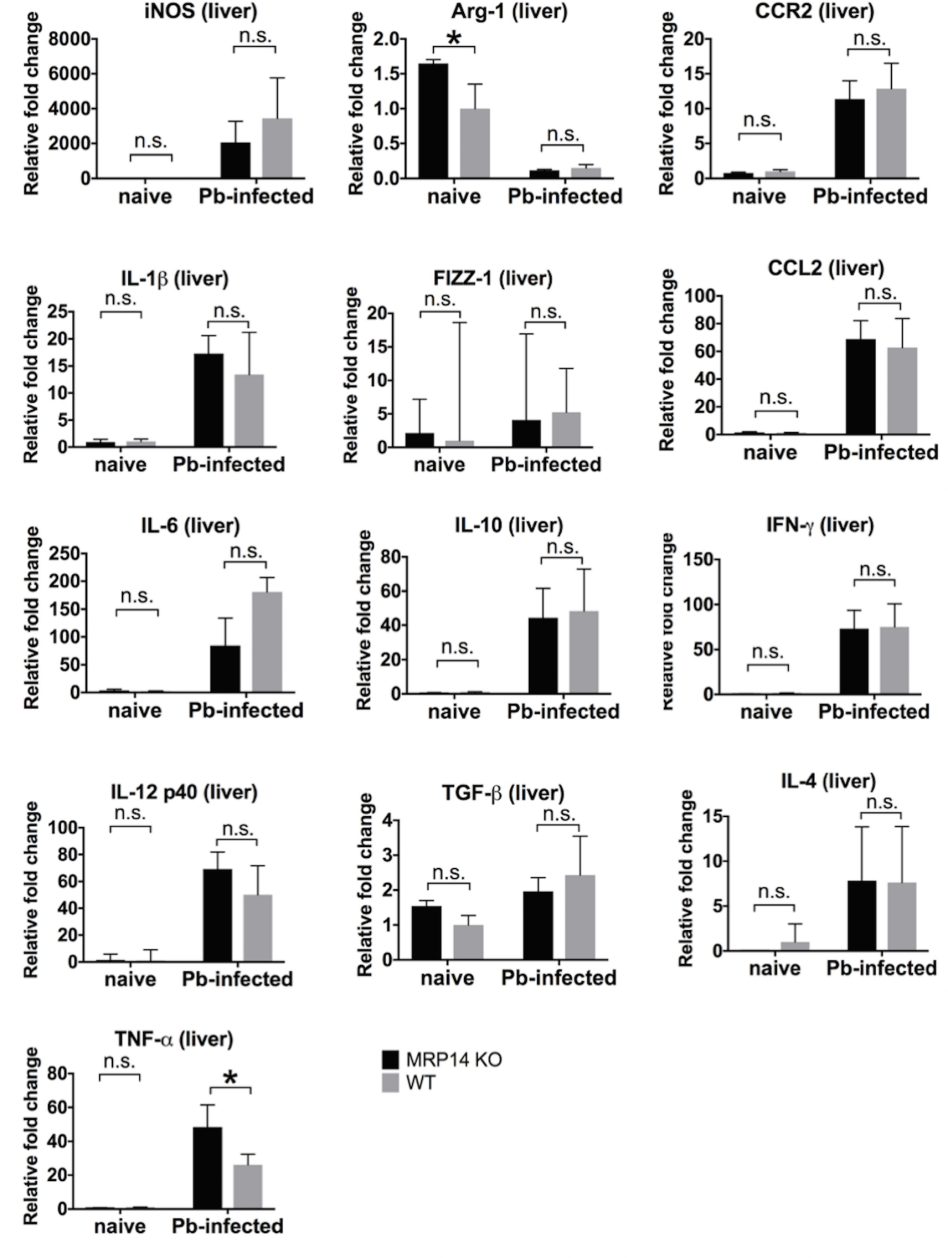
Enhanced pro-inflammatory molecules independent of MRP14 in the liver during rodent malaria. mRNA expression of iNOS, IL-1β, IL-6, IL-12 p40, TNF-α, Arg-1, FIZZ-1, IL-10, TGF-β, NOX2, CCR2, CCL2, IFN-γ and IL-4 in the liver of MRP14-KO mice and WT mice after Pb-infection (n = 5). Graphs show mean and SD of each group. Data are representative of two independent experiments. ^*^P < 0.05; n.s., not significant.

Quantitative RT-PCR analysis showed that the expression of pro-inflammatory molecules in the liver was comparable between MRP14-KO mice and WT mice. In the liver on day 7 post-infection, the expression of iNOS, IL-1β, IL-6, IL-12 p40, TNF-α, IL-10, TGF-β, CCR2, CCL2, IFN-γ and IL-4 was significantly up-regulated, and the expression of Arg-1 was down-regulated in both of MRP14-KO mice and WT mice (Fig 9). In Pb-infected liver, the expression level of iNOS, IL-1β, IL-6, IL-12 p40, Arg-1, FIZZ-1, IL-10, TGF-β, CCR2, CCL2, IFN-γ and IL-4 was not significantly different between MRP14-KO mice and WT mice, though TNF-α was slightly up-regulated in MRP14-KO mice.

## Discussion

Previous reports showed that T cells and IFN-γ play a critical role in the protective immunity against non-lethal murine malarial strain, *P*. *chabaudi* or *P*. *berghei* XAT [27–29]. On the other hand, other reports demonstrated that T cells and IFN-γ are potentially involved in the pathogenesis during lethal rodent malarial strain, *P*. *berghei* NK65 or *P*. *berghei* ANKA [9,30,31]. In order to elucidate whether T cells are involved in the pathogenesis of hepatic injury during infection with *P*. *berghei* ANKA, T cell-deficient *nu/nu* mice were infected with Pb-pRBCs. It is revealed that hepatic injury during Pb-infection was induced through T cell-dependent mechanism regardless of parasite number. Cell stimulation assay indicates that Pb-pRBC antigen-specific T cells play a pivotal role in IFN-γ and iNOS production (Fig 1F and 1E). Besides, even though the number of MRP14^+^ cells was comparable between *nu/nu* mice and WT mice, the secretion of MRP14 is dependent on T cells (Figs 1D and 2A). The mechanism of MRP14 secretion has been unclear, and our study revealed that T cell is associated to one side of the MRP14 secretion mechanism. There was a positive correlation between serum concentration of hepatic enzymes and MRP14 (Figs 1D and 2A). This relation between hepatic injury and serum MRP14 level indicates that extracellular MRP14 has some functions involved in the pathogenesis of hepatic injury during rodent malaria. Considering that both MRP14^+^ cells and T cells were present with the increased number in the liver during Pb-infection (Fig 2C), it is suggested that MRP14 assists the function of T cells to induce nitric oxide (NO) through IFN-γ in the liver during rodent malaria. We observed that rMRP14 promoted NO production from RAW264.7 cells stimulated with IFN-γ (data not shown), which suggests that MRP14 potentiates IFN-γ-induced NO production. Because high levels of NO, generated primarily by iNOS, have cytotoxic and pro-inflammatory effects leading to severe hepatic injury [32,33], MRP14 seems to promote hepatic injury by increasing NO production during rodent malaria. Interestingly, MRP14^+^ cells were accumulated in the liver during Pb-infection in both of *nu/nu* and WT mice, suggesting a T cell-independent mechanism for accumulating MRP14^+^ cells during Pb-infection.

Cell stimulation assay demonstrated that MRP14 is an agonist of TLR2 and TLR4 (Fig 3). TLRs are a class of transmembrane proteins that play important roles in the inflammatory responses. When activated by agonists, TLRs signal cascade leads to the activation of the transcription factor NF-*κ*B and induces cytokine expression and secretion [34,35]. Actually, rMRP14 induced pro-inflammatory molecules such as IL-1β, TNF-α, IL-6, CCL2 and iNOS from macrophages (Fig 3), whose expression was induced by downstream signaling of TLR. Polymyxin B, LPS inhibitor, did not suppress rMRP14 activity, indicating that LPS contamination is excluded (Fig 3B). Recombinant EGFP, which was produced with the same expression/purification method as rMRP14, showed no activity to RAW264.7, indicating that other compartments of bacteria are not contaminated in the recombinant proteins. There is a report that MRP14 induces the secretion of TNFα, IL-1β and IL-6 dependent on TLR4 [36], and it is also reported that these inflammatory cytokines can be associated with the pathology during infection with *P*. *berghei* [37]. In this study, we showed that MRP14 promotes the secretion of pro-inflammatory cytokines such as IL-1β, TNF-α, IL-6 and IL-12 from macrophages via TLR2 and TLR4. Since IL-12 is a potent inducer of IFN-γ, it is considered that MRP14 promote IL12-induced IFN-γ production from Pb-pRBC antigen-specific T cells, which facilitates the production of NO. Accordingly, it is hypothesized that MRP14-activated macrophages are involved in T cell activation. Considering that cytokines act in small environment and that MRP14^+^ macrophages were co-existed with T cells (Fig 2C), the interaction between MRP14^+^ macrophages and T cells by cytokines support our hypothesis. Taken together, MRP14 can be involved in the pathology of hepatic injury during malaria through activation of TLR2 and TLR4 signaling. It is reported that MRP8 also acts as an endogenous activator of TLR4 and promotes inflammatory processes in infection and autoimmunity by amplifying TNF-α release in response to LPS [38,39]. Besides, TLRs recognize not only damage-associated endogenous molecular patterns (DAMPs) but also pathogen-associated molecular patterns (PAMPs) [40], and promotes inflammatory responses against pathogens. In malaria, it is known that hemozoin as malaria pigment activates innate immune response through TLR9 [41]. TLR-TLR cross-talk induces the synergistic effect. It was reported that the stimulation with MALP2 and LPS (TLR2 and TLR4 ligands, respectively) results in the production of TNF-α at levels much greater than that observed for each of the ligands alone [42]. Besides, TLR4 and TLR9 were shown to synergize in the production of TNF-α in macrophages in a manner associated with enhanced MAPK signaling [43]. To take those facts into account, the activation of TLR2 and TLR4 by MRPs as DAMPs can amplify immune response with TLR9 activation by hemozoin as PAMPs in malaria. Moreover, considering that TLRs are expressed in various cells such as macrophages and dendritic cells, the increase of MRP14 in plasma may be involved in the systemic inflammation through TLRs in malaria.

In order to verify whether extracellular MRP14 is involved in the pathology of hepatic injury during malaria, Pb-infected mice were intravenously injected with rMRP14. Because MRP14 can form heterodimer with MRP8 [15], it is anticipated that the concentration of MRP14 measured by sandwich ELISA can be lower than the actual MRP14 concentration. Accordingly, the dose of rMRP14 (50 μg) for administration was set based on the western blot analyses for MRP14 levels in serum of Pb-infected mice as previously reported [26]. The rMRP14 dose was consistent with the dose in other studies about MRP14 administration [38,44–46]. We confirmed that serum MRP14 was significantly elevated after rMRP14-injection (S1 Fig). Even though the number of parasites was comparable between rMRP14-injected mice and PBS-injected control mice, extracellular MRP14 exacerbated hepatic injury along with enhanced serum AST and ALT during rodent malaria (Fig 4). Actually, in human cases, it is reported that there was a significant correlation between plasma MRP8/MRP14 levels and liver damage as illustrated by elevated ALT levels of patients infected with *Salmonella typhi* [47], which suggests that extracellular MRP14 is involved in hepatic injury. In this study, we did not measure the quantity of liver parasites, assuming that blood parasite load can reflect the liver parasite burden. However, it is still possible that MRP14 affects liver pathology by manipulating local parasite burden. This point should be addressed in future experiments in order to elucidate the association of liver parasite level with hepatic injury through MRP14-dependent mechanisms. Immunohistochemical analysis demonstrated that extracellular MRP14 promotes the accumulation of MRP14^+^ cells in the liver during Pb-infection (Fig 5A and 5C). The accumulation level and staining pattern of MRP8^+^ cells and MRP14^+^ cells were similar (data not shown). The accumulation of MRP14^+^ especially in focal necrosis areas suggests that MRP14 can be involved in the inflammation and necrosis of hepatic cells (Fig 5F). Because the number of peripheral leukocytes also increased in rMRP14-injected mice (Fig 5E), these accumulated MRP14^+^ and MRP8^+^ cells in the tissue seem to be recruited from bone marrow. Because the number of the accumulated MRP14^+^ and MRP8^+^ cells was not changed after perfusion (data not shown), it is considered that the MRP14^+^ and MRP8^+^ cells were adherent to endothelial cells in the sinusoid of liver. Also, even in the absence of Pb-infection, administration of rMRP14 could induce the accumulation of MRP14^+^ and MRP8^+^ cells in the tissue of naïve mice (Fig 5B and 5D), which indicates that MRP14^+^ and MRP8^+^ cells can be accumulated by MRP14 alone. We observed that 50% of total macrophages phagocytized hemozoin in HE-stained tissue, and that most of the hemozoin-phagocytizing macrophages showed rich deposition of hemozoin in big cytoplasm. On the other hand, hemozoin-phagocytizing MRP14^+^ macrophages were rarely observed (S2 Fig). The deposition level of hemozoin in the MRP14^+^ macrophages was low and the cytoplasm of MRP14^+^ macrophages is small. If phagocytes uptake rMRP14, rich deposition of hemozoin in big plasma is supposed to be observed in MRP14^+^ macrophages, too. Thus, the results indicate that MRP14-positive stain is not caused by phagocytosis of rMRP14 uptake. Quantitative RT-PCR analysis revealed that extracellular MRP14 induces the up-regulation of pro-inflammatory molecules such as iNOS, IL-1β, IL-12 p40, TNF-α and NOX2 in the liver of naïve mice (Fig 6), which is consistent with the result *in vitro* (Fig 3). iNOS, which induces high concentrations of NO, and is transcriptionally regulated and induced by inflammatory cytokines, i.e., IL-1β, TNF-α or IFN-γ [48,49]. Because those cytokines and iNOS were elevated after Pb-infection (Fig 7), it is indicated that the pro-inflammatory cytokines and NO induced by MRP14 are involved in the inflammatory response, which leads to hepatic injury during rodent malaria. Other studies have shown that NO and iNOS are involved in hepatic injury [50,51]. Also in malaria, NO and iNOS have been implicated in the pathogenesis during malaria such as hypotension, immunosuppression, and cerebral malaria [52–54]. NOX2, which is expressed in macrophages, is also important to generate superoxide, which reacts with NO and leads to cytotoxic peroxynitrite [55]. Although rMRP14-injected mice showed a trend towards an increase in inflammatory molecules compared with PBS-injected controls during Pb-infection, we found no significant differences among the two groups. Time point may be one of the factors which induced no significant differences in inflammatory markers between rMRP14-injected mice and PBS-injected mice. It is considered that the production of inflammatory molecules is saturated on day 7 post-infection. Hence, we hypothesize that inflammatory markers are promoted significantly at earlier time point (for example on day 3 post-infection) in rMRP14-injected mice than PBS-injected mice. In the absence of Pb-infection, extracellular MRP14 alone could not induce hepatic injury illustrated by elevated AST and ALT levels (Fig 4). Since rMRP14 administration did not influence the expression of IFN-γ in naïve mice (Fig 6), and increase rate of iNOS expression induced in Pb-infected mice was much higher than that induced by rMRP14 alone (Figs 6 and 7), IFN-γ produced by Pb-specific T cells have a potent role in iNOS induction and hepatic injury. Also, extracellular MRP14 induced the up-regulation of chemotactic factors, CCR2 and CCL2, which suggests that these molecules are associated with the cellular recruitment to the tissue during rodent malaria. Actually, it is reported that MRP can induce migration of neutrophils and monocytes [56–59]. It is also reported that monocytes migrated by CCR2 and CCL2 aggravate hepatic injury [60,61]. Taken together, these data indicate that elevated MRP14 during malaria is one of the key molecules for pathology of hepatic injury, and it is concluded that extracellular MRP14 promotes the accumulation of MRP14^+^ and MRP8^+^ cells in the liver and the local production of pro-inflammatory molecules, which leads to hepatic injury during rodent malaria.

Because blood level of MRP14 was high in *P*. *berghei*-infected mice (~4 µg/ml by sandwich ELISA, Fig 2), *in vivo* neutralization using commercial antibodies or inhibitors may not be realistic. Therefore, we utilized MRP14-KO BALB/c mice [62] in order to know the function of extracellular MRP14 in the pathogenesis of hepatic injury during rodent malaria. Although it was revealed by the rMRP14 administration experiment that extracellular MRP14 exacerbates hepatic injury during rodent malaria, improvement of liver inflammation during Pb-infection could not been observed in MRP14-KO mice. The MRP14-KO mice also showed hepatic injury during Pb-infection, and their serum AST and ALT levels were comparable to WT controls (Fig 8A), indicating that hepatic injury was not improved in MRP14-KO BALB/c mice during rodent malaria. Besides, there was no significant difference in pro-inflammatory cytokines levels in the liver between MRP14-KO mice and WT mice, i.e., iNOS, IL-1β, IL-12, IFN-γ, CCR2 and CCL2 (Fig 9). The results indicate that MRP14 deficiency did not affect the induction of those pro-inflammatory molecules during rodent malaria. Serum MRP8 levels in the infected MRP14-KO mice were comparable to that in the infected WT mice (Fig 8F). Also, quantitative analysis revealed the number of MRP8^+^ macrophages in the liver was comparable between MRP14-KO BALB/c mice and WT mice after Pb-infection (Fig 8D). Although rMRP14 administration exacerbates liver inflammation and hepatic injury, MRP14 deficiency could not influence the levels of hepatic injury and pro-inflammatory molecules during Pb-infection. We previously reported that bone marrow cells from MRP14-KO BALB/c mice secreted higher TNF-α upon stimulation with LPS than WT controls, and that MRP14-KO mice showed comparable inflammatory cytokine levels to WT mice in LPS-induced shock model [62]. Our previous results suggest that MRP14 deficiency induces not only the simple deletion of extracellular MRP14 function to promote inflammation but also affects intracellular MRP14 function to maintain TLR4 signaling in myeloid cells. It is considered that intracellular MRP14 deficiency induces the hyperresponsiveness of TLR4 signaling in myeloid cells. The expression level of inflammatory cytokines in the liver was comparable between MRP14-KO and WT mice during Pb-infection, which may be explained by the higher reactivity of TLR4 signaling in BMCs of MRP14-KO BALB/c mice. Actually, GPI anchor molecules of *Plasmodium* parasites as well as LPS is known to activate TLR4 as a PAMPs [63]. Thus, our results suggest that MRP14-KO mice were not the optimal model to analyze extracellular MRP14 function as an inflammatory enhancer. In addition, some studies reported that MRP14 is dispensable for liver inflammation in several models [47,64,65]. In a murine *Salmonella* infection model, though elevated serum MRP14 and the accumulation of MRP14^+^ and MRP8^+^ cells in the liver were observed, MRP14 deficiency did not influence bacterial growth, liver damage and mortality [47]. Also, in the carbon tetrachloride-induced liver inflammation model, liver inflammation, fibrosis and recruitment of inflammatory cells were not affected upon MRP14 deletion [65]. Those reports are consistent with our results in a Pb-infection model.

Taken together, MRP14 function in hepatic injury during rodent malaria is hypothesized as below. The secretion of MRP14 is dependent on T cells after Pb-infection. Extracellular MRP14 promotes the secretion of pro-inflammatory cytokines such as IL-1β, TNF-α, IL-6 and IL-12 from macrophages via TLR2 and TLR4. The pro-inflammatory cytokines induce the up-regulation of iNOS, which produces high concentration of NO. NOX2, which is expressed in macrophages, is also important to generate superoxide, which reacts with NO and leads to cytotoxic peroxynitrite. High levels of NO have cytotoxic and pro-inflammatory effects leading to necrosis of hepatocytes. IL-12 is a potent inducer of IFN-γ, and IFN-γ produced by Pb-pRBC antigen-specific T cells (presumably Th1 differentiation) will increase iNOS expression in the macrophages through M1 polarization, which facilitates the production of NO. Extracellular MRP14 also promotes the accumulation of macrophages (including MRP8^+^ and MRP14^+^ macrophages) in the sinusoid of liver, to which CCR2 and CCL2 may contribute. In this way, the accumulated macrophages promote the inflammatory cascade by positive feedback of cytokines and further macrophage recruitment, which leads to hepatic injury.

Thus, the present study revealed that extracellular MRP14 is one of key molecules for liver inflammation during rodent malaria. It is concluded that extracellular MRP14 promotes inflammatory reactions through the accumulation of MRP14^+^ macrophages and the up-regulation of pro-inflammatory cytokines and NO, which exacerbates hepatic injury during rodent malaria. MRP14 deficiency induces not only the simple deletion of extracellular MRP14 function to promote inflammation but also induces the hyperresponsiveness of TLR4 signaling in myeloid cells, which keep comparable inflammatory cytokine levels to WT during inflammation.

## Materials and methods

### Animals

BALB/c mice and BALB/c-nu/nu (nu/nu) mice were purchased from Japan Clea, and all mice were acclimated for 1 week. Mice were maintained under specific pathogen-free conditions in a temperature- and humidity-controlled room under a 12-h light/dark cycle with unrestricted access to food and water. MRP14-KO BALB/c mice (BALB/cAJcl-S100a9<em1Fjw>, [62]) were bred in the animal facility at the Graduate School of Agricultural and Life Sciences, The University of Tokyo. The male mice were used for experiments at the age of 8–9 weeks. The animal experiments were reviewed and approved by an institutional animal research committee and an institutional committee on genetically modified organisms at the Graduate School of Agricultural and Life Sciences, The University of Tokyo. Animal health and well-being was assessed in accordance with the Guidelines for Proper Conduct of Animal Experiments (the Science Council of Japan) and the National Institutes of Health guidelines for the use of experimental animals. No animal died prior to the end of experiment.

### Experimental infection and hematological analysis

Experimental infection was performed as previously described [26]. Briefly, to prepare Pb-pRBCs, blood was collected from a BALB/c mouse infected with *P*. *berghei* ANKA and was mixed well with citrate-phosphate-dextrose as anticoagulant. The RBCs were washed two times with Hanks’ Balanced Salt Solution (Life Technologies, Carlsbad, CA) by centrifugation at 400 x*g* for 5 min. Mice were infected intraperitoneally with 10^6^ Pb-pRBCs and sacrificed at day 7 of infection. For administration of rMRP14, mice were injected with rMRP14 (50 µg/mouse) or PBS intravenously every day for 7 days with or without Pb-infection. Recombinant proteins used in this study were produced as previously described [62]. An endotoxin level of the recombinant proteins was measured by LAL endotoxin assay (GenScript USA Inc., Piscataway, NJ) and shown to be below 100 EU/mg of protein. Blood samples were collected every day by cutting the tip of tail to monitor parasitemia level. One-thousand RBCs (including pRBCs) were counted on Giemsa-stained thin blood smears for each mouse by microscopic examination. Mice were anaesthetized under isoflurane vapor, and whole blood was collected by cardiac puncture of mice and centrifuged for 10 min at 5,000 x*g* to collect serum. To analyze the inflammatory responses to Pb-infection in vivo, the liver, spleen and serum were collected from mice at day 7 of infection. For hematological analysis, blood was collected using the heparinized capillary tubes (TERUMO, Tokyo, Japan) and hematocrit was determined by centrifuging the tubes at 15,000 x*g* for 10 min. For quantitative analyses of leukocytes, peripheral leukocytes were counted with Türk’s solution (Merck Millipore, Darmstadt, Germany).

### Determination of cytokines and hepatic enzymes

Hepatic enzymes, AST and ALT, were measured by Japan Society of Clinical Chemistry standard method using automatic analyzer, Hitachi 7189 (Hitachi, Tokyo, Japan). MRP14 or MRP8 concentration in serum and TNF-α and IFN-γ in culture supernatants were measured by using commercial sandwich ELISA kit (R&D systems, Minneapolis, MN for MRP14 and MRP8; eBioscience, San Diego, CA for TNF-α and IFN-γ).

### Nitric oxide (NO) measurement

NO production was assessed by measuring the accumulation of nitrites in the cell culture medium using the colorimetric Griess reaction. Culture medium was mixed with Griess reagent (1% sulfanilamide, 0.1% N-1-naphthyl-ethylendiamide and 2.5% phosphoric acid) in 1:1 ratio. After incubation for 10 min, the optical density (OD) was read at 550 nm on an absorbance detector. Standard curve was generated using NaNO_2_ to determine the quality of NO_2_^-^.

### HE-staining and immunohistochemical analyses

HE staining and immunohistochemical staining was performed as previously described [26]. Briefly, for immunohistochemical staining, paraffin-embedded tissues, sectioned at 4 µm thickness, were dewaxed and boiled in Tris-EDTA buffer (10 mM Tris Base, 1mM EDTA-2Na, 0.05% Tween 20, pH 9.0) for 20 minutes. After blocking, anti-MRP8 (Santa Cruz Biotechnology, Santa Cruz, CA), anti-MRP14 (Santa Cruz Biotechnology), anti-CD3 antibody (Santa Cruz Biotechnology) or anti-CD45R (BD Biosciences, San Jose, CA) was applied to the serial sections of tissues. After washing with PBS, sections were incubated with biotinylated anti-goat IgG (Nichirei Bioscience, Tokyo, Japan) or biotinylated anti-rat IgG (Nichirei Bioscience), followed by incubation with alkaline phosphatase-conjugated streptavidin (Nichirei Bioscience). Finally, enzymatic color development was performed by using 4-[(4-amino-m-tolyl) (4-imino-3-methylcyclohexa-2,5-dien-1-ylidene)methyl]-o-toluidine monohydrochloride (new fuchsine, Nichirei Bioscience). For quantitative analyses of infiltrating MRP14^+^ and MRP8^+^ cells in the tissues, the number of MRP14^+^ or MRP8^+^ cells in the immunohistochemically stained tissues was counted in 5 random microscopic fields at 400x magnification.

### Cell stimulation assay

The murine macrophage cell line RAW264.7 was purchased from American Type Culture Collection, Manassas, VA. RAW264.7 were grown in DMEM culture medium (Sigma-Aldrich, St Louis, MO) supplemented with 10% fetal bovine serum (Thermo Fisher Scientific, Waltham, MA), 100 U/ml penicillin and 100 μg/ml streptomycin (P/S; Life Technologies), at 37°C in 5% CO_2_. RAW264.7 (2 x 10^5^ cells /ml) were applied and incubated in 96-well plates. 24 hr later, cells were treated with MRP14 (5 µg/ml), MRP8 (5 µg/ml) or lipopolysaccharide (LPS) (5 ng/ml, from *E*. *coli* 055:B5, Sigma-Aldrich) incubated with or without IFN-γ (20 ng/ml, PeproTech, Rocky Hill, NJ). After incubation for 3–48 hr, RNA was extracted with TRIzol (Thermo Fisher Scientific.). MRP8 and LPS were used as a positive control. Polymyxin B (50 µg/ml) (Sigma-Aldrich) was used as a LPS inhibitor. Paquinimod (250 µg/ml) was used as a MRP14 inhibitor, and was gently provided from Active Biotech, Lund, Sweden. EGFP is recombinant proteins with polyhistidine-tag and was used as negative control.

Single suspension of splenocytes were collected by passing the tissue through a 70 µm cell strainer (BD Biosciences). Erythrocytes were lysed with Red Blood Cell Lysing buffer Hybri-MAX^TM^ (Sigma-Aldrich) for 2 min at room temperature, and remaining cells were washed three times with PBS. Splenocytes (5 x 10^6^ cells/ml) were cultured in RPMI 1640 medium (Sigma-Aldrich) supplemented with 10% FBS (Thermo Fisher Scientific), 100 U/ml penicillin and 100 μg/ml streptomycin (Life Technologies), at 37°C in 5% CO_2_. Splenocytes were treated with Pb-pRBCs (1 x 10^8^ cells/ml), naïve RBC (nRBC) (1 x 10^8^ cells/ml) or concanavalin A (conA: 3 µg/ml, Sigma-Aldrich). After incubation for 24 hr, supernatants were collected for determination of cytokine concentrations.

TLR ligand screening assay was performed by InvivoGen, San Diego, CA to assess whether MRP14 activates TLR pathways. TLR stimulation was tested by assessing NF-*κ*B activation in HEK293 cells, which express one of murine TLRs (TLR2, 3, 4, 5, 7, 8 and 9). The SEAP reporter is under the control of promoter inducible by the NF-*κ*B. This reporter gene allows the monitoring of signaling through the TLRs, based on the activation of NF-*κ*B. In a 96-well plate containing 2.5 x 10^5^ cells/ml, rMRP14 (5 μg/ml) was added. After incubation for 24 hr, cells activation was evaluated as an increase in SEAP activity measured as absorbance at OD (650 nm) on an absorbance detector.

### Quantitative RT-PCR

RNA was extracted and cDNA was synthesized by reverse transcription. Tissues were homogenized with 1 ml TRIzol (Thermo Fisher Scientific) and φ1.0 stainless steel beads in the 2 ml tube using Micro Smash MS100R (TOMY, Tokyo, Japan) at 4°C. After transferred to the Eppendorf tube, 0.2 μl chloroform was added and centrifuged at 12,000 x*g* for 15 min at 4°C. The supernatant was mixed with 0.5 ml 2-propanol and centrifuged at 12,000 x*g* for 15 min at 4°C. After washing with ethanol, RNA was dissolved in UltraPure distilled water (Thermo Fisher Scientific). The concentration of total RNA was measured by DU 730 Life Science UV/vis spectrophotometer (Beckman Coulter, Brea, CA), and 400 ng of total RNA was used as the template for the synthesis of 20 µl cDNA. The mixture including 1.25 µM oligo (dT)_16_, and 0.5 mM dNTPs (Thermo Fisher Scientific) with template RNA in the tube was incubated for 5 min at 65°C. After adding 5x first strand buffer and 10 mM DTT (Thermo Fisher Scientific), 200 U M-MLV (Thermo Fisher Scientific) was added and the tube was incubated for 50 min at 37°C and 15 min at 70°C. cDNA was synthesized and analyzed for expression of cytokines (primers are listed in S1 Table). Real-time polymerase chain reaction (PCR) assay was carried out using 2 μl of cDNA as the template and 10μl of SYBR Select Master Mix (Thermo Fisher Scientific) on the ABI Prism 7000 Sequence Detection System (Thermo Fisher Scientific). Data was analyzed by 2^-ΔΔCt^ methods and normalized by GAPDH. The thermal cycling conditions for the PCR were 94°C for 10 min, followed by 40 cycles of 94°C for 15 sec and 60°C for 1 min.

### Statistical analysis

Statistical analysis was performed using GraphPad Prism 7.0 software package (GraphPad Software Inc., San Diego, CA). Results are presented as mean + standard deviation (SD). The differences between the groups of mice were analyzed by two-way ANOVA followed by Sidak multiple comparisons test. Student’s *t* test was used to compare the differences in the results from two independent groups. *P* value less than 0.05 were considered significantly different. In order to compare the variation of samples rather than to estimate population mean, we used SD instead of SEM.

### Ethics statement

All animal experiments were reviewed and approved by the Animal Experiment Committee at the University of Tokyo (Approval No. P14-943, P15-91, 764–2630, 830–2630). The experiments were performed in accordance with the Regulations for Animal Care and Use of the University of Tokyo, which were based on the Law for the Humane Treatment and Management of Animals, Standards Relating to the Care and Management of Laboratory Animals and Relief of Pain (the Ministry of the Environment), Fundamental Guidelines for Proper Conduct of Animal Experiment and Related Activities in Academic Research Institutions (the Ministry of Education, Culture, Sports, Science and Technology) and the Guidelines for Proper Conduct of Animal Experiments (the Science Council of Japan). Collection of peripheral blood was performed under anesthesia with isoflurane. At the end of the experiments, the animals were euthanized by exsanguination under anesthesia with isoflurane followed by cervical dislocation.

## Supporting information

S1 Fig Serum MRP8 and MRP14 concentration of rMRP14-injected mice. Serum concentration of MRP8 (left) and MRP14 (right) of mice injected with rMRP14 or PBS after Pb-infection (n = 5). Graphs show mean and SD of each group. Data are representative of two independent experiments. ^*^P < 0.05.
(TIF)

S2 Fig Low phagocytic activity of MRP14^+^ macrophages. (a) The rate of hemozoin (malaria pigment)-phagocytosis. In HE-stained, MRP14-stained and MRP8-stained tissues, the number of hemozoin-phagocytizing macrophage was counted in 5 random microscopic fields at x 400 magnification. The ratio of hemozoin-phagocytizing macrophage to total macrophages, the ratio of hemozoin-phagocytizing MRP14^+^ macrophage to total MRP14^+^ macrophages, and the ratio of hemozoin-phagocytizing MRP8^+^ macrophage to total MRP8^+^ macrophages were expressed as percentage. MΦ, macrophage. Graphs show mean and SD of each group. Data are representative of two independent experiments. ^*^P < 0.05. (b) Representative hemozoin-phagocytizing macrophage and MRP14^+^ macrophage. In HE-stained tissue, most of hemozoin-phagocytizing macrophages showed rich deposition of hemozoin in cytoplasm. Hemozoin-phagocytizing MRP14^+^ macrophages and MRP8^+^ macrophages were rarely observed, and the deposition of hemozoin in the MRP14^+^ macrophages and MRP8^+^ macrophages was small. Bar, 5 μm.
(TIF)

S1 Table Primer list. (PDF)
