# Supplementary figures and images for: Exacerbation of hepatic injury during rodent malaria by myeloid-related protein 14
Source: PLoS One. 2018 Jun 14;13(6):e0199111. doi: 10.1371/journal.pone.0199111 (PMC6002122; doi:10.1371/journal.pone.0199111)

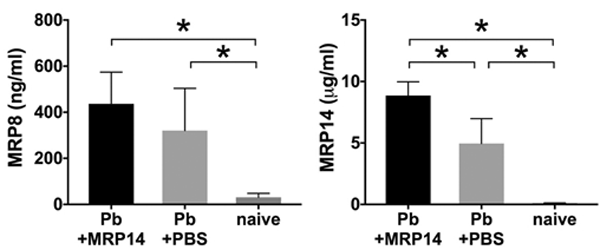

Supplement: S1 Fig — Serum concentration of MRP8 (left) and MRP14 (right) of mice injected with rMRP14 or PBS after Pb-infection (n = 5). Graphs show mean and SD of each group. Data are representative of two independent experiments. *P < 0.05. (TIF) [file pone.0199111.s001.tif]

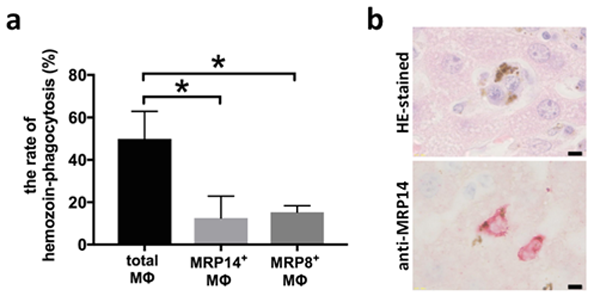

Supplement: S2 Fig — (a) The rate of hemozoin (malaria pigment)-phagocytosis. In HE-stained, MRP14-stained and MRP8-stained tissues, the number of hemozoin-phagocytizing macrophage was counted in 5 random microscopic fields at x 400 magnification. The ratio of hemozoin-phagocytizing macrophage to total macrophages, the ratio of hemozoin-phagocytizing MRP14+ macrophage to total MRP14+ macrophages, and the ratio of hemozoin-phagocytizing MRP8+ macrophage to total MRP8+ macrophages were expressed as percentage. MΦ, macrophage. Graphs show mean and SD of each group. Data are representative of two independent experiments. *P < 0.05. (b) Representative hemozoin-phagocytizing macrophage and MRP14+ macrophage. In HE-stained tissue, most of hemozoin-phagocytizing macrophages showed rich deposition of hemozoin in cytoplasm. Hemozoin-phagocytizing MRP14+ macrophages and MRP8+ macrophages were rarely observed, and the deposition of hemozoin in the MRP14+ macrophages and MRP8+ macrophages was small. Bar, 5 μm. (TIF) [file pone.0199111.s002.tif]
