# Supplementary material for: Exacerbation of hepatic injury during rodent malaria by myeloid-related protein 14
Source: PLoS One. 2018 Jun 14;13(6):e0199111. doi: 10.1371/journal.pone.0199111 (PMC6002122; doi:10.1371/journal.pone.0199111)
Supplement: S1 Table — (PDF) [file pone.0199111.s003.pdf]

S1 Table. Primer list

Gene	Forward	Reverse
IL-1β	5′-GAAAGACGGCACACCCACCCT-3′	5′-GCTCTGCTTGTGAGGTGCTGATGTA-3′
IL-6	5′-CCAGAGATACAAAGAAATGATGG-3′	5′- ACTCCAGAAGACCAGAGGAAAT-3′
IL-12 p40	5′-ACAGCACCAGCTTCTTCATCAG-3′	5′-TCTTCAAAGGCTTCATCTGCAA-3′
TNF-α	5′-CTGTGAAGGGAATGGGTGTT-3′	5′-GGTCACTGTCCCAGCATCTT-3′
iNOS	5′-GTTCTCAGCCCAACAATACAAGA-3′	5′-GTGGACGGGTCGATGTCAC-3′
Arg-1	5′-CTCCAAGCCAAAGTCCTTAGAG-3′	5′-AGGAGCTGTCATTAGGGACATC-3′
FIZZ-1	5′-CCAATCCAGCTAACTATCCCTCC-3′	5′-CCAGTCAACGAGTAAGCACAG-3′
IL-10	5′-GCTGGACAACATACTGCTAACC-3′	5′-CCCAAGTAACCCTTAAAGTCCTG-3′
TGF-β	5′-GTCAGACATTCGGGAAGCAG-3′	5′-GCGTATCAGTGGGGGTCA-3′
CCR2	5′-TGCCATCATAAAGGAGCCAT-3′	5′-TTTGTTTTTGCAGATGATTCAA-3′
CCL2	5′-GTTGGCTCAGCCAGATGCA-3′	5′-AGCCTACTCATTGGGATCATCTTG-3′
IFN-γ	5′-GGCCATCAGCAACAACATAAGCG-3′	5′-TGGGTTGTTGACCTCAAACTTGG-3′
IL-4	5′-GGCATTTTGAACGAGGTCAC-3′	5′-AAATATGCGAAGCACCTTGG-3′
GAPDH	5′-CGACTTCAACAGCAACTCCCACTCTTCC-3'	5′-TGGGTGGTCCAGGGTTTCTTACTCCTT-3′
β-actin	5′-GTTACCAACTGGGACGACA-3′	5′-TGGCCATCTCCTGCTCGAA-3′
NOX2	5′-AGCTATGAGGTGGTGATGTTAGTGG-3′	5′-CACAATATTTGTACCAGACAGACTTGAG-3′
